# Perinatal Mental Health Among Forced Migrant Women: A Scoping Review of Prevalence and Associated Factors

**DOI:** 10.3389/ijph.2026.1608845

**Published:** 2026-03-24

**Authors:** Yamma Khalid Aria, Amanda Mason-Jones, Ada Keding

**Affiliations:** Department of Health Sciences, University of York, York, United Kingdom

**Keywords:** anxiety, depression, forced migration, perinatal mental health, social support

## Abstract

**Objectives:**

To synthesise existing evidence on the prevalence of perinatal mental health disorders among forced migrant women and to identify factors influencing mental health outcomes in this population.

**Methods:**

A scoping review was conducted following the Arksey and O’Malley framework. Electronic databases and grey literature sources were searched for studies published in English between 1951 and August 2022. Studies were included if they examined perinatal mental health among forced migrant women and clearly distinguished forced from voluntary migration.

**Results:**

A total of 1,105 records were identified, of which 16 studies met the inclusion criteria (12 quantitative, three qualitative, and one mixed-methods study). Two main themes emerged: the prevalence of perinatal mental health disorders, including depression, anxiety, and post-traumatic stress disorder, and factors influencing mental health outcomes, such as social support, exposure to traumatic events, and stigma.

**Conclusion:**

Forced migrant women experience a high burden of perinatal mental health difficulties. Improved clarity in migration definitions and consistency in outcome measurement may strengthen future research and support more effective responses to perinatal mental health needs in this population.

## Introduction

Forced migration exposes those experiencing it to various risks that can lead to poor health outcomes either in their host country or during transit to another country [1]. These risks include lack of social support leading to social isolation, family separation, trauma, gender-based violence, torture, rape, and challenges related to language, culture, and legal status [2–5]. Such experiences have important implications for mental health, particularly for women, who constitute a substantial proportion of forced migrants and often face gender-specific vulnerabilities.

The perinatal period, defined as pregnancy and up to 1 year after childbirth, represents a critical window for mental health. Mental health disorders during this period remains high across populations and have been associated with adverse consequences for both maternal and child health [6, 7]. For forced migrant women, perinatal mental health may be shaped by the cumulative effects of pre-migration trauma, migration-related stressors, and post-migration social and structural challenges.

A growing body of literature has examined mental health outcomes among migrant populations, including women of reproductive age. However, studies vary considerably in how migrant populations are defined, often grouping together voluntary and forced migrants despite important differences in migration trajectories, exposures, and legal contexts. In addition, perinatal mental health outcomes among forced migrant women have been investigated using diverse study designs, measurement tools, and conceptual frameworks, making it challenging to synthesise evidence and draw overarching conclusions.

Scoping reviews are well suited to mapping heterogeneous bodies of literature, clarifying key concepts, and describing how research in a given field has been conducted [8]. Within this context, the present scoping review maps the existing evidence on perinatal mental health among forced migrant women, focusing on the prevalence of depression, anxiety, and post-traumatic stress disorder, as well as the risk and protective factors examined in the literature. This review aims to describe the scope and characteristics of the available evidence, identify dominant themes, and highlight areas that were less frequently examined within this body of research.

## Methods

This scoping review was conducted using the methodological framework proposed by Arksey and O’Malley (2005), which is commonly applied to map the extent, nature, and characteristics of research activity within a field [9]. The reporting of this review was informed by the Preferred Reporting Items for Systematic Reviews and Meta-Analyses extension for Scoping Reviews (PRISMA-ScR) [10].

The review followed five stages as outlined by Arksey and O’Malley: (1) identifying the research questions; (2) identifying relevant studies; (3) study selection; (4) charting the data; and (5) collating, summarising, and reporting the results.

### Search Strategy

A comprehensive search of electronic databases was conducted, including Embase, PubMed, PsycINFO, PsycArticles, CINAHL, the Maternity & Infant Care Database, and Google Scholar. These databases were selected to capture a broad range of medical, psychological, and public health literature relevant to perinatal mental health.

The final database search was conducted up to the 1st of August 2022, and appropriately tailored search strategies were developed for each database to ensure all relevant studies were captured. In addition to peer-reviewed literature, the search also included evidence from grey literature sources. Databases such as OpenGrey (https://www.opengrey.eu/) and Mednar (https://mednar.com/) as well as a Google Search (examining at least the first ten pages), were searched to identify any relevant grey literature. Reference lists of included studies and citation index searches were also examined to identify additional relevant publications.

The search strategy combined three core concepts: forced migration, the perinatal period, and mental health. For forced migration, a combination of free-text terms and truncation was used, including “forced migration,” “displacement,” “refugee*,” and “asylum seeker*.” The perinatal period was captured using the terms “pregnan*,” “pregnancy,” “perinatal*,” and “perinatal period.” Mental health-related terms included “mental health,” “depression*,” “anxiet*,” “post-traumatic stress disorder,” and “PTSD.” Boolean operators (AND/OR) were used to combine search terms.

### Eligibility Criteria

Studies were eligible for inclusion if they examined mental health outcomes during the perinatal period among women who were forcibly displaced across international borders and identified as refugees or asylum seekers. Studies involving mixed migrant populations were included only when data for refugees or asylum seekers were reported separately or when these groups were included as comparators. Studies focusing exclusively on voluntary or labour migrants were excluded. Studies involving internally displaced persons were excluded due to differences in legal status and migration context.

Quantitative, qualitative, and mixed-methods studies were eligible for inclusion. No restrictions were placed on study design. Studies published from 1951 onwards were included, corresponding to the adoption of the United Nations Convention relating to the Status of Refugees [11]. No restrictions were placed on geographical setting or host country. The search was restricted to studies published in English.

### Study Selection

The study selection process involved screening the titles and abstracts of the identified studies according to the eligibility criteria. Full-text articles for all studies meeting the criteria were retrieved, and further screening was performed. To ensure accuracy and consistency, 10% of the full-text articles were randomly screened by a second reviewer. No disagreements were identified during this process.

### Data Charting

Data were extracted using a standardised data extraction form developed for this review. Extracted information included study characteristics (authors, year of publication, country), participant characteristics (sample size and population description), study design and setting, mental health outcomes assessed, measurement tools used, and key findings related to prevalence and associated factors. Data extraction was conducted by the primary reviewer.

### Data Synthesis

Extracted data were synthesised descriptively. Findings were grouped inductively into thematic categories based on mental health outcomes and associated risk and protective factors identified across studies. No formal appraisal of study quality or certainty of evidence was undertaken, consistent with the aims of a scoping review.

### Ethical Considerations

As this scoping review synthesised data from publicly available studies, formal ethical approval was not required.

## Results

**FIGURE 1:**
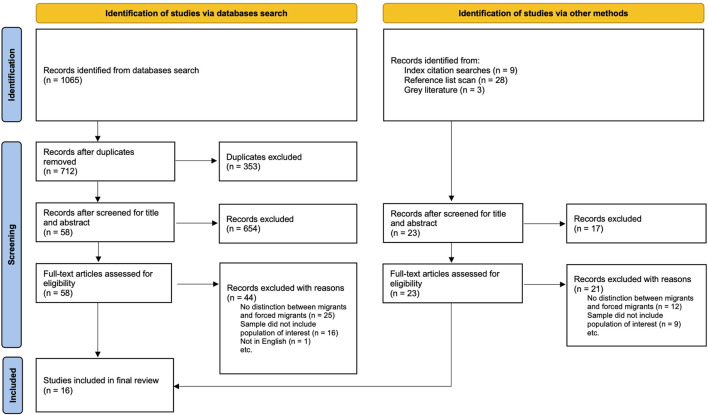
Study selection flow diagram illustrating the selection process of studies included in the scoping review (Global literature, 2008–2022).

In total, 1,105 articles were identified through the search strategy. After removing duplicates, 712 articles were screened by title and abstract, and 81 full-text articles were assessed for eligibility. Ultimately, 16 studies met the eligibility criteria and were included in the review (Figure 1).

Of the included studies, twelve employed quantitative designs, including eight cross-sectional studies [12–19], and four cohort studies [20–23]. One study used a qualitatively driven mixed methods design [24], and three studies employed qualitative methods [25–27].

Studies were conducted across a range of host countries, including Australia (n = 5) [12, 13, 15, 25, 27], Canada (n = 3) [18, 23, 24], the Thai-Myanmar border (n = 3) [21, 22, 26], Turkey (n = 1) [19], Denmark (n = 1) [20], Germany (n = 1) [14], Jordan (n = 1) [16], and Israel (n = 1) [17]. All studies were published between 2008 and 2022 (Table 1).

**TABLE 1:** Characteristics of the studies included in the scoping review (Global literature, 2008–2022).

First author, year	Country	Study design	Sample size	Participant characteristics	Outcome(s)	Measurement tool(s)	Study objective(s)
Ahmed et al. [24]	Canada	Qualitatively driven mixed methods study	12	Refugee women only	Depression, anxiety, post-traumatic stress disorder (PTSD)	- Edinburgh postnatal depression scale (EPDS) - PTSD screening instrument - Qualitative interviews	- How do syrian refugee women view maternal depression - What are the social support needs and challenges faced by them? - What barriers do they encounter when trying to access mental health services? - How common is depression among syrian refugee
Akgor et al. [19]	Turkey	Cross-sectional study	100	Refugees vs. host-born women	Depression	- Beck’s depression inventory (BDI)	- To compare the psychiatric profiles and self-concept of Turkish pregnant adolescents with those of refugee pregnant adolescents - To evaluate the factors associated with psychiatric problems among refugees
Blackmore et al. [12]	Australia	Cross-sectional study	52	Refugee women only	Depression, anxiety, PTSD	- EPDS (validated Dari version) - Diagnostic interview	- To examine the screening effectiveness of the EPDS - To assess the EPDS’s ability to identify depressive and anxiety disorders in refugee populations
Blackmore et al. [13]	Australia	Cross-sectional study	52	Refugee women only	PTSD, subthreshold PTSD	- Harvard trauma questionnaire (HTQ)	- To explore the symptomology and diagnoses of PTSD and subthreshold PTSD. - To assess the screening effectiveness of HTQ
Castaner et al. [20]	Denmark	Register-based cohort study	274,916	Refugee women and Danish women	Perinatal psychiatric episode	- Danish national health registers (psychiatric diagnoses)	- To compare psychiatric morbidity during the perinatal period between refugees and Danish-born women, including predictors of psychiatric morbidity
Dennis et al. [23]	Canada	Prospective cohort study	1,125	Refugee and asylum seeking women vs. non-refugee immigrant and Canadian-born women	Depression	- EPDS	- To examine the prevalence and persistence of post-partum depression (PPD) - To evaluate the sensitivity, specificity, and predictive accuracy of the EPDS
Fellmeth et al. [21]	Thai Myanmar border	Prospective cohort study	451	Refugee women vs. labour migrants	Depression	- Structured clinical interview for Diagnosis (SCID) - Social support scale	- To assess the relationship between social support and perinatal depression
Fellmeth et al. [22]	Thai Myanmar border	Prospective cohort study	567	Refugee women vs. labour migrants	Depression	- EPDS - Social support measures	- To determine the prevalence of, and factors contributing to, perinatal depression
Fellmeth et al. [26]	Thai Myanmar border	Qualitative	11	Refugee women vs. labour migrants	Depression	- In-depth qualitative interviews	- To gain a deeper insights into the experiences of severe perinatal depression among refugee and labour migrant women
Kaufmann et al. [14]	Germany	Cross-sectional study	120	Refugee women only	Depression, anxiety, PTSD	- EPDS - Gad-7 - PC-PTSD - PHQ-2	- To highlight the characteristics and needs of refugee women
Mohammad et al. [16]	Jordan	Cross-sectional study	365	Refugee women only	Depression	- EPDS	- To investigate the prevalence of PPD and its associated risk factors
Nakash et al. [17]	Israel	Cross-sectional study	38	Asylum seeking women only	Depression	- EPDS	- To examine the association between postnatal depression and acculturation
Nithianandan et al. [27]	Australia	Qualitative	37	Refugee women and health professionals	Depression, anxiety, PTSD	- Semi-structured qualitative interviews	- To examine the barriers and facilitators for implementing perinatal mental health screening
Rees et al. [15]	Australia	Cross-sectional study	1,335	Refugee women vs. Australian-born women	Major depressive disorder	- Mini-international neuropsychiatric interview (MINI)	- To identify the psychosocial risk factors and prevalence of major depressive disorder (MDD)
Stewart et al. [18]	Canada	Cross-sectional study	341	Refugee and asylum seeking women vs. immigrants and Canadian-born women	Depression	- EPDS	- To investigate the prevalence of PPD and PPD symptoms in newcome women
Willey et al. [25]	Australia	Qualitative	22	Refugee and asylum seeking women vs. migrant women	Depression, anxiety	- Qualitative interviews - Screening program records	- To assess whether the screening program improved detection of depression and anxiety symptoms compared to routine care

The included studies assessed mental health outcomes during pregnancy and/or up to 1 year postpartum. Outcomes most frequently examined were depression, anxiety, and post-traumatic stress disorder (PTSD), measured using a range of screening instruments and diagnostic interviews. Variation in measurement tools and cut-off thresholds was observed across studies.

The findings of this review are structured around two main themes: (1) the prevalence of mental health disorders among forced migrant women, and (2) factors (risk or protective) influencing mental health outcomes.

### The Prevalence of Mental Health Disorders Among Forced Migrant Women

#### Depression

The prevalence of depression during the perinatal period was examined in ten studies [12, 14–19, 21, 23, 24]. Across these studies, the reported prevalence of perinatal depression among forced migrant women ranged from approximately 17% to over 80%. In all studies that included comparison groups, the prevalence of depression was higher among forced migrant women than among non-migrant women.

Several studies used the Edinburgh Postnatal Depression Scale (EPDS), with a high proportion of participants scoring above the depression cut-off, although the thresholds applied differed across studies. Nakash et al. found that 81.6% of Eritrean asylum seekers in Israel scored above an EPDS cut-off of 13, with a mean score of 14.9 (SD = 5.5) [17]. Ahmed et al. reported that 58% of Syrian refugee women in Canada exceeded an EPDS cut-off of 10, while Mohammad et al. found that 49.6% of Syrian refugee women in Jordan scored above 12 on the EPDS, with a mean score of 12.6 (SD = 3.3) [16]. Differences in EPDS thresholds limited direct comparability across studies but consistently indicated a substantial burden of depression among forced migrant women.

Studies including non-migrant comparison groups similarly demonstrated higher rates of perinatal depression among forced migrant women. Stewart et al. reported postpartum depression in 25.7% of refugees and 31.1% of asylum seekers, compared with 8.1% of Canadian-born women [18]. Blackmore et al. identified current depression in 25% of Afghan refugee women attending an Australian antenatal clinic, based on a validated Dari version of the EPDS with a cut-off score greater than nine [12].

Longitudinal evidence suggested persistence of depression beyond the immediate postpartum period. Dennis et al. reported that elevated EPDS scores were present among refugees and asylum seekers at both 1 and 16 weeks postpartum [23]. They found that the prevalence of EPDS >9 at 1 and 16 weeks postpartum was 26.6% and 18.2% for refugees, and 25.2% and 24.1% for asylum seekers, respectively. In contrast, only 14.8% and 7.4% of Canadian-born women had postpartum depression at 1 and 16 weeks postpartum, respectively. Similarly, the prevalence of EPDS >12 at 1 and 16 weeks postpartum was 10.5% and 11.2% for refugees, and 10.8% and 14.4% for asylum seekers, respectively. Whereas for Canadian-born women, it was 4.8% and 2.9%, respectively [23].

Other measurement instruments yielded similar findings. Using Beck’s Depression Inventory (BDI), Akgor et al. reported that 28% of pregnant refugee adolescents in Turkey scored above the clinical cut-off of 17, compared with 12% of Turkish-born adolescents [19]. Rees et al. found that 32.5% of refugee women met criteria for major depressive disorder using a structured diagnostic interview, compared with 14.5% of Australian-born women [15]. Along the Thai-Myanmar border, Fellmeth et al. reported that 47.3% of refugee women and 38.6% of migrant women met criteria for perinatal depression using the Structured Clinical Interview for the Diagnosis (SCID) [21]. Although Kaufmann et al. reported a comparatively lower prevalence of 17.5% among refugee women in a German reception centre, depression remained more common in this population than in general perinatal populations [14].

#### Anxiety

The prevalence of anxiety during the perinatal period was examined in three studies [12, 14, 24]. Across these studies, the reported prevalence of anxiety among forced migrant women ranged from 15% to 50%, depending on the measurement tool and cut-off thresholds used.

Two studies assessed anxiety using anxiety subscales of the Edinburgh Postnatal Depression Scale (EPDS), applying different cut-off thresholds. Blackmore et al. reported that 21% of Afghan refugee women exceeded an anxiety subscale threshold of 4 (mean = 5.82, SD = 0.75), whereas Ahmed et al., using a lower threshold of 3 on the same subscale, found that 50% of Syrian refugee women screened positive for anxiety (mean = 3.6, SD = 2.5) [12, 24]. Differences in thresholds likely contributed to variation in reported prevalence across studies.

Kaufmann et al. assessed anxiety using the GAD-7 and reported that 15% of refugee women met the cut-off score of 10 or higher, indicating clinically significant anxiety. Although prevalence estimates varied across studies, all findings indicated elevated levels of anxiety among forced migrant women when compared with reported estimates for general perinatal populations (8.5%–10.5%) [28–30].

#### Post-Traumatic Stress Disorder (PTSD)

Post-traumatic stress disorder during the perinatal period was examined in three studies [13, 14, 24]. Reported prevalence of PTSD among forced migrant women ranged from approximately 6%–36%.

Kaufmann et al. reported the highest prevalence, with 36.1% of refugee women diagnosed with PTSD [14]. Ahmed et al. found that 17% of Syrian refugee women exceeded the relevant cut-off score for PTSD [24]. Blackmore et al. using the Harvard Trauma Questionnaire, reported a lower prevalence of 5.8% among refugee women [13]. However, the study noted a substantial proportion of women experienced subthreshold PTSD symptoms, demonstrating that many women still faced considerable psychological trauma despite not meeting the full diagnostic criteria for PTSD [13].

### Factors Influencing Mental Health Outcomes

Across the included studies, several factors were reported to influence perinatal mental health outcomes among forced migrant women. Three key themes emerged: social support, exposure to traumatic events including violence and abuse, and stigma and barriers to accessing mental health services. These factors reflected experiences occurring before, during, and after migration, as well as during pregnancy and the postpartum period.

#### Social Support

Social support was consistently identified as a major protective factor for perinatal mental health across quantitative and qualitative studies [15, 16, 18–22]. Lower levels of perceived or actual support were associated with higher risks of depression and anxiety among forced migrant women. Some works [21, 22] highlighted that perceived support was a stronger predictor of mental health outcomes than actual amount of support received.

Quantitative findings showed that insufficient perceived social support was consistently associated with poorer perinatal mental health outcomes among forced migrant women. In studies conducted along the Thai-Myanmar border, Fellmeth et al. reported that women with low perceived social support had statistically significantly higher odds of perinatal depression (OR = 1.89, 95% CI 1.10–3.24) [21]. Similar findings were reported in an earlier study by Fellmeth et al. where insufficient perceived social support more than doubled the odds of perinatal depression (OR = 2.10, 95% CI = 1.10–3.70) [22]. Mohammad et al. also demonstrated a statistically significant association between low levels of social support and depression among Syrian refugee women in Jordan, reporting a moderate positive correlation between lower social support scores and higher EPDS scores (r = 0.44, p-value <0.01) [16].

Comparative studies further indicated that forced migrant women experienced lower levels of social support than non-migrant women, alongside higher rates of perinatal depression. Stewart et al. reported markedly lower social support scores among refugees and asylum seekers compared with Canadian-born women [18], while Rees et al. observed that women who had fewer than two confidants were more susceptible to major depressive disorder (aOR = 1.62, 95% CI 1.18, 2.23) [15]. Partner support emerged as a particularly important aspect of social support [19, 20]. Castaner et al. found that refugee women living without a partner were 1.5 times more likely to experience a perinatal psychiatric episode (PPE) compared with those living with a partner (95% CI 1.36–1.65). Similarly, Akgor et al. reported that living without a partner was a statistically significant predictor of higher depression scores among both refugee women (p = 0.023) and Turkish-born women (p = 0.001) during the perinatal period [19].

Qualitative evidence [24, 27] supported the quantitative findings by describing how separation from family and limited social networks contributed to emotional distress during pregnancy and postpartum. Participants linked depressive symptoms to *“being away from her family, alienation”* [24] and emphasised the importance of close female relatives as a source of reassurance and practical support around childbirth: *“when you are going to give birth, you really need your mother, your aunt or somebody”* [24]. Reuniting with family members was described as a key way to reduce distress and restore a sense of support; one woman explained that being able to visit relatives, or for relatives to visit them, would prevent feeling *“like a bird in a cage or imprisoned”* [24]. Similarly, Nithianandan et al. reported that community representatives emphasised family support as a protective factor and identified lack of social support as a major risk factor for mental health deterioration among refugee women [27].

#### Traumatic Events Including Violence and Abuse

Four quantitative studies [13, 15, 21, 22] reported that exposure to traumatic events (TEs), including violence and abuse was associated with poorer perinatal mental health outcomes among forced migrant women. Across these studies, cumulative trauma exposure was linked to higher odds of perinatal depression and PTSD. For example, Blackmore et al. reported that women with PTSD had higher trauma scores than women without PTSD [13], indicating a relationship between trauma burden and PTSD symptoms during the perinatal period.

Along the Thai-Myanmar border, Fellmeth et al. found that women who reported traumatic events had increased odds of perinatal depression; among refugees, the adjusted odds of perinatal depression were 1.63 times higher compared with those who did not report traumatic events (aOR = 1.63, 95% CI 1.23–2.17) [21]. Similar findings were reported in an earlier study by Fellmeth et al. where women with a history of trauma had increased odds of perinatal depression (OR = 2.17, 95% CI 1.38–3.41) [22].

Rees et al. reported higher exposure to TEs and interpersonal violence (IPV) among refugee women compared with Australian-born women. In this study, 29.8% of refugee women reported two or more traumatic events compared with 19.0% of Australian-born women (p < 0.001), and 42.9% of refugee women reported IPV compared with 20.5% of Australian-born women (p < 0.001) [15]. Furthermore, trauma and violence exposure were associated with increased likelihood of major depressive disorder (MDD), including among women reporting two or more TEs (aOR = 2.13, 95% CI 1.47–3.08), severe psychological IPV (aOR = 1.62, 95% CI 1.18–2.23), and physical IPV (aOR = 4.64, 95% CI 2.78–7.74) [15].

Qualitative findings further described the persistence of interpersonal violence and abuse within intimate relationships during pregnancy and postpartum, ranging from emotional abuse to physical violence and sexual coercion [26]. Participants described experiences of criticism and intimidation, for example: “[My husband] says that I am not a good mother… it hurts me so much that I would cry” [26], as well as severe physical violence: “He beat me up and squeezed my neck…” [26]. Accounts also included sexual coercion within relationships: “I was 18 or 19 years old, He was kissing and hugging me in front of people… so I had to marry him” [26].

#### Stigma and Barriers to Accessing Mental Health Services

Three qualitative studies reported that stigma and fear of judgment acted as major barriers to help-seeking for mental health concerns among forced migrant women during the perinatal period [24, 25, 27]. Across these studies, women expressed concerns about being labelled as *“crazy,”* experiencing shame, or being perceived negatively by family members and the wider community if they disclosed mental health difficulties or sought professional support.

In Ahmed et al. stigma was one of the most frequently cited barriers to accessing mental health services. One participant explained, *“I might have depression… If I went to a psychiatrist, they would say that she went crazy”* [24]. Although some women indicated a willingness to seek care despite anticipated stigma: *“I’m sure that they will speak about me and say that I’m crazy but this doesn’t matter to me”* [24], these accounts highlighted the emotional and social costs associated with disclosure.

Similarly, Willey et al. found that stigma could prevent follow-up even when women initially agreed to a mental health referral. As one participant described, *“Most of them feel embarrassed… They avoid going because they think it affects their dignity”* [25]. In addition to community-level stigma, concerns about confidentiality emerged as a barrier in relation to the use of interpreters. Nithianandan et al. reported that some women felt uncomfortable discussing mental health concerns in the presence of interpreters, although female interpreters and healthcare professionals were often perceived as more trustworthy: *“With a female interpreter, we feel safe and we feel comfortable so everything that we wanted to express…we can say it easily”* [27]. In contrast, participants in Ahmed et al (2017) described interpreter involvement as unacceptable due to fears of breaches of confidentiality: *“If you speak with a psychiatrist… but if there is an interpreter … this person might speak about what you said….”* [24].

Overall, these qualitative findings indicated that stigma surrounding mental health, combined with concerns about confidentiality and dignity, substantially limited forced migrant women’s engagement with perinatal mental health services.

## Discussion

This scoping review synthesised evidence on the prevalence and factors associated with perinatal mental health disorders among forced migrant women. Across the included studies, forced migrant women consistently experienced a higher burden of depression, anxiety, and post-traumatic stress disorder compared with non-migrant populations.

The elevated prevalence of perinatal mental health disorders observed in this review is consistent with findings from previous reviews examining migrant populations more broadly. For example, a systematic review by Fellmeth et al. reported substantially higher rates of perinatal depression and anxiety among migrant women compared with host populations [31]. Similarly, a recent review by Stevenson et al. highlighted that migrant women experience disproportionate mental health challenges during pregnancy and the postpartum period, although many studies combined forced and voluntary migrants within a single category [32]. By focusing exclusively on forced migrant women, the present review adds clarity to the evidence-base by demonstrating that elevated risk persists when this group is examined separately.

Exposure to traumatic events (TEs), including violence and abuse, emerged as a central factor associated with poor perinatal mental health outcomes. The findings of this review reinforce existing evidence that cumulative trauma occurring before, during, and after displacement across the migration trajectory substantially increases the risk of depression, anxiety, and PTSD among forced migrant women [33]. Previous reviews have similarly identified trauma exposure as a key determinant of maternal mental health in refugee populations [34]. The quantitative and qualitative evidence included in this review underscores the importance of recognising trauma histories when assessing and supporting perinatal mental health in this population.

Stigma surrounding mental health was consistently identified as a barrier to help-seeking among forced migrant women. Fear of judgement, concerns about confidentiality, and discomfort with interpreter-mediated consultations limited engagement with mental health services. These findings are in line with prior qualitative syntheses highlighting stigma and cultural norms as significant obstacles to care for migrant and refugee women during the perinatal period [35–37]. Together, this evidence suggests that improving access to perinatal mental healthcare requires culturally sensitive, trauma-informed approaches that address both individual and structural barriers.

Social support emerged as a key protective factor across the included studies. Both perceived and actual support from partners, family members, and social networks were associated with better perinatal mental health outcomes. Previous studies have highlighted social support as a central protective factor for maternal mental health among migrant and refugee women [31, 38, 39]. The qualitative evidence further highlights the emotional impact of family separation and the importance of social connectedness during pregnancy and the postpartum period.

### Strengths and Limitations

A key strength of this review is its exclusive focus on forced migrant women, allowing for a clearer examination of perinatal mental health outcomes within this population. In addition, the inclusion of both quantitative and qualitative studies provided a comprehensive overview of prevalence estimates as well as lived experiences and contextual factors.

Several limitations should be acknowledged. The search was restricted to English-language publications, which may have led to the exclusion of relevant studies conducted in other languages. While some evidence from low- and middle-income settings was captured, including studies conducted along the Thai–Myanmar border, the available literature from these contexts remains relatively limited compared with high-income host countries. In addition, as this was a scoping review, no formal appraisal of study quality was undertaken.

### Implications for Future Research

Future research may benefit from building on the areas identified in this review by further examining perinatal mental health among forced migrant women across a wider range of study designs and contexts. Greater clarity and consistency in the definition of migration status, particularly in distinguishing forced from voluntary migration experiences, would strengthen comparability across studies. In addition, improved transparency and consistency in the reporting of mental health outcomes and measurement approaches would facilitate more meaningful comparisons of findings and support a clearer interpretation of prevalence estimates.

### Conclusions

Forced migrant women experience a disproportionately high burden of perinatal mental health disorders, shaped by trauma exposure, social isolation, stigma, and barriers to care. This review highlights the need for targeted research and services that recognise the unique experiences of forced migrant women during the perinatal period. Addressing these challenges is essential for improving maternal mental health and promoting equity in perinatal care.
